# Cortical Hemodynamic Response and Connectivity Modulated by Sub-threshold High-Frequency Repetitive Transcranial Magnetic Stimulation

**DOI:** 10.3389/fnhum.2019.00090

**Published:** 2019-03-19

**Authors:** Rihui Li, Thomas Potter, Jun Wang, Zhixi Shi, Chushan Wang, Lingling Yang, Rosa Chan, Yingchun Zhang

**Affiliations:** ^1^Department of Biomedical Engineering, University of Houston, Houston, TX, United States; ^2^Guangdong Provincial Work Injury Rehabilitation Hospital, Guangzhou, China; ^3^Department of Computer Science, Sun Yat-sen University, Guangzhou, China; ^4^Department of Electrical Engineering, City University of Hong Kong, Hong Kong, China

**Keywords:** repetitive transcranial magnetic stimulation, near infrared spectroscopy, hemodynamic response, cortical excitability, brain connectivity

## Abstract

Repetitive transcranial magnetic stimulation (rTMS) at sub-threshold intensity is a viable clinical strategy to enhance the sensory and motor functions of extremities by increasing or decreasing motor cortical excitability. Despite this, it remains unclear how sub-threshold rTMS modulates brain cortical excitability and connectivity. In this study, we applied functional near-infrared spectroscopy (fNIRS) to investigate the alterations in hemodynamic responses and cortical connectivity patterns that are induced by high-frequency rTMS at a sub-threshold intensity. Forty high-frequency (10 Hz) trains of rTMS at 90% resting motor threshold (RMT) were delivered through a TMS coil placed over 1–2 cm lateral from the vertex. fNIRS signals were acquired from the frontal and bilateral motor areas in healthy volunteers (*n* = 20) during rTMS administration and at rest. A significant reduction in oxygenated hemoglobin (HbO) concentration was observed in most defined regions of interest (ROIs) during the stimulation period (*p* < 0.05). Decreased functional connectivity within prefrontal areas as well as between symmetrical ROI-pairs was also observed in most participants during the stimulation (*p* < 0.05). Results suggest that fNIRS imaging is able to provide a reliable measure of regional cortical brain activation that advances our understanding of the manner in which sub-threshold rTMS affects cortical excitability and brain connectivity.

## Introduction

Transcranial magnetic stimulation (TMS) is a therapeutic technique that applies a rapid alternating current to generate magnetic fields, which penetrate scalp and skull to reach the brain and induce secondary electrical fields that activate the cortical neurons (Wassermann and Lisanby, 2001). When TMS is provided periodically at a specific frequency, it is referred to as repetitive TMS (rTMS; Kobayashi and Pascual-Leone, 2003). Specifically, rTMS inhibits cortical excitability at lower pulse frequencies (less than the 1 Hz) and enhances the cortical excitability at higher pulse frequencies (5 Hz or higher; Ridding and Ziemann, 2010; Valero-Cabré et al., 2017). Since its introduction in 1985 (Barker et al., 1985), rTMS has demonstrated the ability to induce long-term changes in cortical activity (Fitzgerald et al., 2006b), leading to its adoption as a potential treatment for a variety of neurological and mental disorders, including depression and motor impairment (Takeuchi et al., 2005; Fitzgerald et al., 2006a; O’Reardon et al., 2007; Schwerin et al., 2011). The underlying mechanism of action for rTMS, however, remains poorly understood. In order to maximize the therapeutic potential of rTMS, it is necessary to investigate the effects that stimulation has on both brain activity and cortical connectivity.

To date, a number of neuroimaging modalities have been used to investigate the functional changes associated with TMS. The most dominant technological investigations have combined TMS with functional MRI (fMRI; Bohning et al., 1999; Bestmann et al., 2008; Wang et al., 2017), electroencephalography (EEG; Bonato et al., 2006; Pigorini et al., 2011; Chung et al., 2015), and magnetoencephalography (MEG; Shibasaki, 2008), or functional near infrared spectroscopy (fNIRS; Mochizuki et al., 2006; Koenraadt et al., 2011; Cao et al., 2013). Each modality has its strengths and weaknesses. FMRI, EEG, and MEG all measure primary or secondary electromagnetic signals that are associated with various brain activities. The magnetic and electrical fields produced by TMS may interfere with these measurements and introduce noise and error to assessments of TMS response. FNIRS, on the other hand, is an emerging noninvasive optical imaging technique that offers an alternative way to investigate TMS-evoked brain activity (Boas et al., 2004). It estimates activity-linked changes in tissue hemoglobin (Hb) concentration based on the different absorption coefficients for oxygenated hemoglobin (HbO) and deoxygenated hemoglobin (HbR; Bunce et al., 2006; Ernst et al., 2012). fNIRS devices with electromagnetism-compatible designs do not interfere with the electromagnetic fields produced by TMS. Consequently, fNIRS holds several advantages over other imaging modalities: a high signal-to-noise ratio, non-interference with electromagnetic fields, portability, and reduced sensitivity to motion artifacts (Li et al., 2017). These features make fNIRS a promising tool to investigate the cortical activation and connectivity associated with rTMS (Kozel et al., 2009; Cao et al., 2013; Groiss et al., 2013).

Previous studies have demonstrated the feasibility of using fNIRS to characterize the effects of rTMS on the cortical excitability and connectivity. Mochizuki et al. examined the HbO, HbR, and total hemoglobin (HBT) change when 1 Hz rTMS was delivered to the motor cortex at 100%, 120%, and 140% of the active motor threshold (AMT, defined as the lowest TMS intensity necessary to induce reliable motor evoke potentials in the target muscle during slightly active contraction; Mochizuki et al., 2006). Results suggested that hemodynamic responses to lower stimulation intensities showed no significant change, while hemodynamic responses at 140% of AMT revealed a significant reduction in both HbR and HbT. Another study focusing on the effects of 1 Hz rTMS on non-motor areas (prefrontal cortex) demonstrated that there was no significant change induced by TMS at 90% and 110% of the resting motor threshold (RMT, defined as the lowest TMS intensity necessary to induce reliable motor evoke potentials in the target muscle at resting), while a significant drop in HbO was induced by 130% RMT stimulation (Thomson et al., 2011b). The impact of rTMS on the cortical connectivity has also been investigated in previous studies. Kozel et al. (2009) measured the fNIRS signals from ipsilateral and contralateral brain regions, during which subjects underwent stimulation (1 Hz, 120% RMT) of the motor cortex and, subsequently, the prefrontal cortex. They reported that stimulation to either the motor and prefrontal cortices resulted in a significant reduction in HbO concentration in both the ipsilateral and contralateral cortices, and the ipsilateral and contralateral changes showed high temporal consistency.

While the majority of previous studies reported the observable regional cerebral blood flow (rCBF) at specific brain regions during rTMS intervention at supra-threshold intensity, the broad impact of sub-threshold rTMS stimulation on multiple brain regions has not been well documented. This may be due to the mild effect of sub-threshold rTMS on brain activation as well as the variation in analytical approaches (stimulation parameters and data analysis methods; Noguchi et al., 2003). In addition, it remains unknown whether sub-threshold rTMS has a significant effect on the connectivity between multiple brain regions. This presents a clear need to explore the full extent of hemodynamic responses to TMS at sub-threshold intensities.

In this study, we aimed to investigate the effects of high-frequency (10 Hz) sub-threshold rTMS on brain cortical excitability and connectivity across multiple brain regions. The global hemodynamic trend of the brain was monitored and change in regional connectivity was evaluated by statistically testing the Pearson correlation coefficients before and during the rTMS intervention. We hypothesized that alterations in brain excitability and connectivity can be induced in multiple cortical regions by high-frequency rTMS at sub-threshold intensity. We also expected that the quantified index of hemodynamic responses proposed in this study could be adjusted to optimize the setting during rTMS treatment and translated to clinical applications.

## Materials and Methods

### Participants

Twenty healthy, right-handed volunteers (16 male, four female, age of 25.65 ± 1.69) were recruited from the local community to take part in this study. All participants were free of current neurological or psychiatric disorders. None of the participants had previous experience with the study paradigm employed in this study. Each subject was fully informed about the purpose and risks of the research, and provided written, informed consent prior to the start of the experiment. All protocols and procedures were approved by the local ethics committee (Guangdong Provincial Work Injury Rehabilitation Center, China) and performed in accordance with the Declaration of Helsinki.

### Concurrent fNIRS/rTMS Configuration

**Figure 1:**
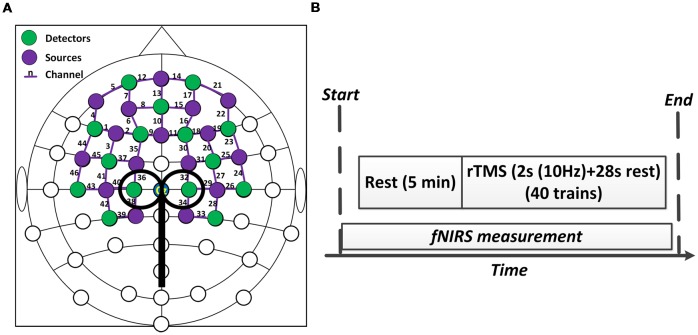
Experiment setup. **(A)** The configuration of concurrent functional near-infrared spectroscopy (fNIRS)-repetitive transcranial magnetic stimulation (rTMS) measurement. **(B)** The protocol of the rTMS. The whole stimulation period lasted about 20 min.

A concurrent fNIRS-rTMS measurement setup was employed in this study. A near infrared spectroscopy system (NIRx Medical Technologies, LLC, Glen Head, NY, USA) consisting of 16 emitters and 15 detectors was utilized to measure hemodynamic responses during rTMS administration. An fNIRS channel was defined as a pair comprised of an emitter and a detector, and all emitters and detectors were mounted by plastic grommets and holders in an elastic cap according to the international 10–20 EEG placement system, as shown in Figure 1A. The inter-optode distance was about 3 cm and a total of 46 fNIRS channels were equidistantly distributed throughout the frontal and bilateral motor areas and were steadily held in place with a plastic strap (Figure 1A). The wavelengths used for the detection of the concentration changes of HbO and HbR were 760 and 850 nm, and all fNIRS signals were acquired at a sampling rate of 3.91 Hz.

**Figure 2:**
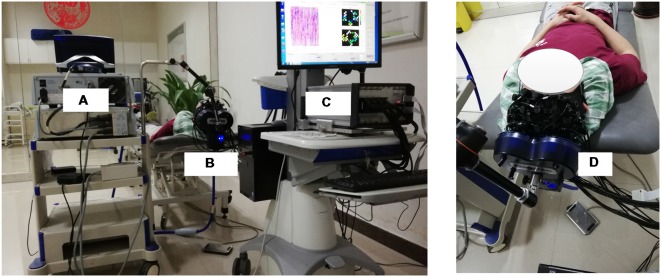
The overview of the whole experimental scheme, including the TMS machine **(A)**, TMS coil **(B,D)** and fNIRS system **(C)**. The subject included in the figure provided written, informed consent for the publication of this figure.

A MagStim Super Rapid magnetic stimulator (MagStim Company, Whitland, Wales, UK) equipped with a commercially available 70-mm diameter figure-eight coil was used to perform rTMS in this study. As shown in Figures 1A, 2, the TMS coil was steadily held and fixed by a metal arm over 1–2 cm lateral from the vertex (Cz channel) of the participant as they lay in a supine position, aligning with the leg representation of the motor cortex (M1; Groppa et al., 2012). For both motor threshold determination and subsequent rTMS, magnetic stimulation was delivered by steadily placing the TMS coil over the fNIRS probes.

### RTMS Protocol

The RMT of each subject was measured and determined prior to the experiment. The RMT was defined as the lowest stimulus intensity that generated motor evoked potentials (MEPs) in the right tibialis anterior muscle (TAM) that were larger than 50 microvolts in 5 of 10 consecutive stimulations (Groppa et al., 2012). The intensity of rTMS in this study was then set at 90% of the RMT intensity. As shown in Figure 1B, the formal experimental session began with a 5 min baseline measurement, during which the rTMS coil was set up and placed over 1–2 cm lateral from the vertex without stimulation delivery. After the baseline measurement, sub-threshold rTMS was performed. Forty trains of TMS were administered over 20 min at 90% RMT. Each train lasted for 30 s and consisted of a 2 s stimulation at 10 Hz frequency and a 28 s break period. FNIRS data were simultaneously collected throughout the whole session, including the baseline and stimulation period. The rTMS protocol used in this study was a clinically standard protocol approved by the collaborated hospital. A complete paradigm of the experiment setup is illustrated in Figure 2.

### Data Preprocessing

In this study, the global temporal change in the hemodynamic properties induced by rTMS was investigated. The global trend was analyzed based on the time course of measurements over the whole TMS experiment (20 min). Acquired fNIRS data were processed and analyzed for each individual subject using MATLAB (2014a, MathWorks, Natick, MA, USA). Considering the global trend was an extremely slow fluctuation (<0.01 Hz), raw fNIRS data were first filtered with a 4th order Butterworth low-pass filter with a high cutoff frequency of 0.2 Hz. In addition, spline interpolation was employed to remove any motion artifact contamination from the fNIRS signal (Scholkmann et al., 2010). The baseline period was defined as the 3 min period immediately before the onset of rTMS administration. Time courses of the changes in HbO, HbR, and HbT concentration throughout the whole stimulation session were calculated relative to the baseline period using the Modified Beer-Lambert Law (Scholkmann et al., 2014). The filtered data were further detrended to remove potential linear drift artifacts from the time series signals.

### Brain Regions Selection

**Figure 3:**
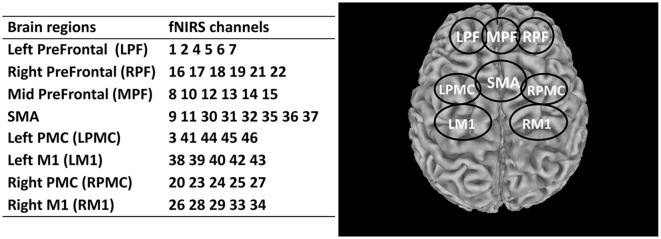
Summary of the segmented regions of interest (ROIs) and assigned fNIRS channels. LPF, Left prefrontal; RPF, Right prefrontal; MPF, Middle prefrontal; SMA, Supplementary motor area; LPMC, Left premotor cortex; RPMC, Right premotor cortex; LM1, Left primary motor cortex; RM1, Right primary motor cortex.

The effects of rTMS on eight regions of interest (ROIs) of the brain were investigated and assessed in this study. These regions included the left prefrontal area (LPF), middle prefrontal area (MPF), right prefrontal (RPF), supplementary motor area (SMA), left premotor cortex (LPMC), right premotor cortex (RPMC), left primary motor cortex (LM1), and right primary motor cortex (RM1). The details of these ROIs and the corresponding fNIRS channels are summarized in Figure 3. A fixed number of fNIRS channels were assigned to each ROI consistently across all subjects.

### Statistical Analysis of Hemodynamic Response

Statistical analysis focused primarily on the HbO signals; while both HbO and HbR were recorded, HbO is considered to be a more robust and sensitive fNIRS parameter than HbR to stimulus-associated changes, and shows stronger correlation with fMRI BOLD response (Plichta et al., 2006; Cui et al., 2011). The signals from the fNIRS channels in each ROI were pooled and averaged to characterize the regional hemodynamic responses. A sliding window approach (60 s window with a 30 s overlap) was then used to smooth the pooled HbO time series of all ROIs for global trend analysis. This method revealed the common temporal pattern of the cortical hemodynamic response by minimizing transient temporal variations. Finally, the regional hemodynamic response induced by rTMS was quantified by calculating the mean area under curve (MAUC) over the entire 20-min pooled HbO time series. The hemodynamic response of the resting condition was also computed as the MAUC within the 3-min baseline period. The difference of MAUC between the two conditions at each region was subsequently tested by paired *t*-test with Bonferroni multiple comparison correction (*p*_corrected_ = 0.0063).

### Functional Connectivity Analysis

In addition to exploring the temporal changes in HbO, the changes in regional connectivity induced by rTMS were evaluated *via* correlation analysis. Connectivity was calculated during the 3-min resting period and 20-min stimulation session, aligning with the lengthy analytical periods used in previous studies (Mesquita et al., 2010; Rosenbaum et al., 2017). The Pearson correlation coefficient between each ROI pair was computed from the HbO time courses during both the resting period and stimulation session, respectively, yielding 28 correlation values for each condition and each subject. The differences in regional connectivity between the resting and stimulation conditions were statistically evaluated using a series of two-tailed *t*-tests with Bonferroni multiple comparison correction to reflect the effects of rTMS on regional brain connectivity (*p*_corrected_ = 0.0018). Prior to statistical testing, all Pearson correlation coefficients were transformed using the Fisher z transformation (Fisher, 1915).

## Results

### Global Effect of the rTMS

**Figure 4:**
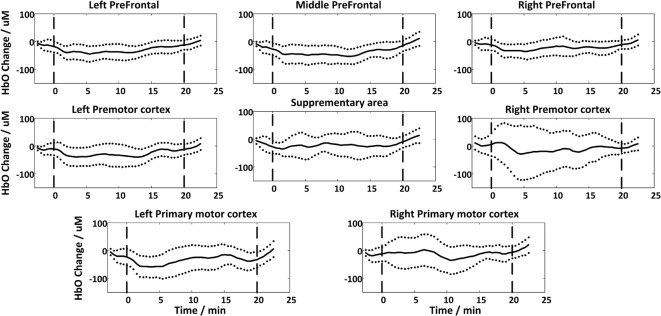
The group-averaged temporal patterns of oxygenated hemoglobin (HbO) concentration changes (black lines) during rest period and the entire 20-min rTMS session in all ROIs. The upper and lower dotted lines denote 95% confidence intervals, while the vertical dash lines denote the start and end of the stimulation.

**Figure 5:**
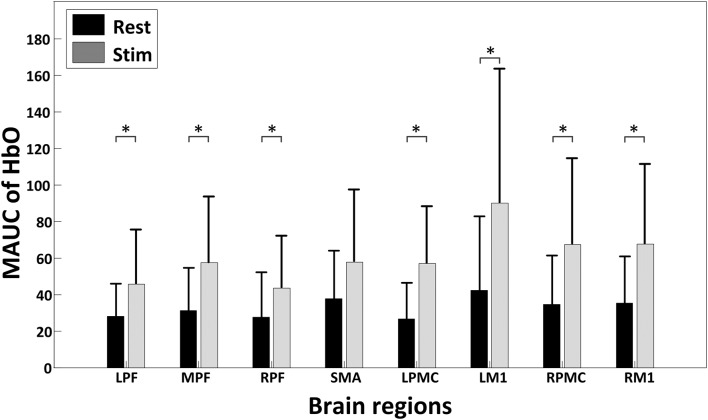
A summary of the mean area under curves (MAUCs) for global temporal HbO concentration changes before and during rTMS. The asterisk “*” indicates a significant difference between the rest period and stimulation session (*p* < 0.05).

Figure 4 shows the global temporal pattern and 95% confidence interval of the HbO concentration in each ROI during the resting period and 20-min rTMS session. As shown in Figure 4, during the rTMS session, the HbO concentrations generally exhibited a noticeable reduction followed by a recovery phase in most ROIs, indicating the effects of rTMS on the hemodynamic response. Statistical testing of the global HbO change (MAUC) revealed a highly significant difference between resting period and the entire 20-min stimulation period in all ROIs (*p* < *p*_corrected_), with the single exception of SMA (*p* = 0.045), as shown in Figure 5.

### Functional Connectivity Analysis

**Figure 6:**
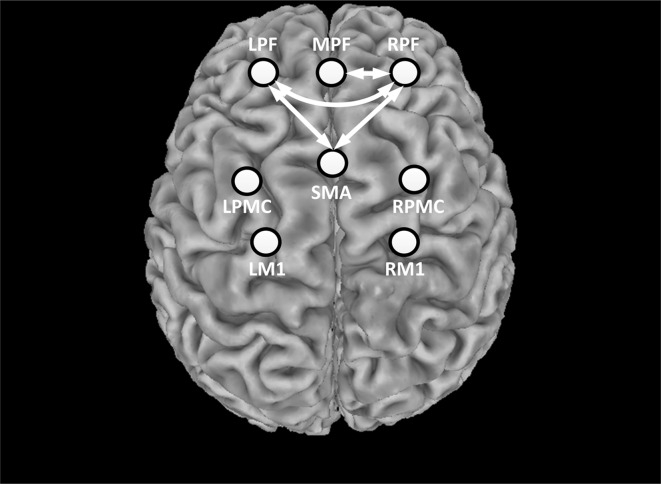
Statistical test results of functional connectivity analysis. The circles denote the defined ROIs, while white arrows denote connections that were significantly decreased during the rTMS session relative to rest period.

Prior to stimulation, the average correlation coefficient of all ROI pairs was 0.674 ± 0.116. During the whole stimulation, however, the average correlation coefficient of all ROI pairs was reduced to 0.615 ± 0.096. This indicated that most ROIs exhibited a decrease in inter-regional connectivity during rTMS administration. The alteration of functional connectivity induced by rTMS was then assessed for each ROI-pair. Figure 6 shows the connections that were significantly suppressed by the rTMS administration. Briefly, the connectivity between the RPF and MPF (*p* = 0.0011) as well as between the RPF and left frontal (LPF; *p* < 0.001) was significantly suppressed by the rTMS, as indicated by the white arrows in Figure 6. In addition to the connections within the prefrontal area, suppression of connectivity was also observed between LPF, RPF and SMA (*p*_LPF-SMA_ = 0.0017, *p*_RPF-SMA_ = 0.001), respectively.

## Discussion

In the current study, the time-sensitive changes in global brain hemodynamics induced by sub-threshold high frequency rTMS were investigated at multiple cortical areas using fNIRS. A significant global reduction of HbO concentration was observed at all selected ROIs except SMA. In addition, the functional connectivity changes induced by rTMS were also examined for each ROI-pair and the pattern of these changes was characterized. The reported results demonstrate the feasibility and capability of using fNIRS to noninvasively assess changes in cerebral hemodynamic responses and inter-regional cortical connectivity during rTMS administration.

The hemodynamic response induced by sub-threshold rTMS in single trial is generally very mild and can be masked when averaging epochs, presenting a challenge to fNIRS-based measurements (Thomson et al., 2011b). In this study, the global changes in HbO concentration were used to investigate the effect of sub-threshold rTMS on the hemodynamic responses and connectivity of the brain. Significant HbO changes were observed in most of the specified brain regions during the rTMS session. The observed regional HbO concentrations typically decreased after the onset of the stimulation and gradually recovered back to the baseline over time, indicating an accumulative effect of the sub-threshold rTMS. This finding is in line with previous fMRI studies, where multiple areas surrounding the stimulated area were deactivated (Jung et al., 2016). A potential explanation for the observed reduction of HbO might be the byproduct effect of rTMS, in which the sensory responses induced by an rTMS pulse may capture the participant’s attention and thereby interrupt normal conscious activity. Another possible explanation for the global reduction in HbO is the concept of “vascular steal”; if the sub-threshold rTMS produced an increase in oxygen consumption at the directly stimulated area that exceeded the local supply, oxygenated blood may have been shunted to the region, resulting in the net drop in the HbO observed at other brain regions (Hu and Huang, 2015). Thomson et al. also reported a similar phenomenon when supra-threshold (120% RMT) 1 Hz rTMS was delivered to PFC, though they only observed a mild early reduction instead of a significant reduction in HbO when a sub-threshold (80% RMT) 1 Hz rTMS was applied (Thomson et al., 2012). This discrepancy may be due to the differences in the stimulus intensity and target location for the studies. A more comprehensive investigation may be necessary to address this in the future.

Interestingly, it is noteworthy that the reduction of the HbO induced by rTMS did not last for the entire rTMS session, as shown in Figure 4. The gradual recovery of the HbO at the end of the rTMS session suggests that the stimulation became less impactful on cortical brain regions as it continued. This may be due to the brain regions gradually adapting to the impact of rTMS or the restoration of regular vascular oxygenation through constant cerebral perfusion. As described in previous studies (Pecuch et al., 2000; Vernieri et al., 2009), vascular innervation represents one of the main regulating factors of cerebral circulation. The activation of the sympathetic nervous system under certain stimulation maintains a constant CBF through vasoconstriction, which might prevent the potential of dilatation of cerebral vessels induced by rTMS in this study. We might hypothesize that long-term rTMS administration might not be beneficial in clinical therapy. However, previous rTMS studies have demonstrated that the effect of stimulation on cortical excitability could linger for a few minutes after stimulation (Romero et al., 2002; Aydin-Abidin et al., 2006). As the direct relationship between hemodynamic response and cortical excitability during TMS administration is absent, whether fNIRS is able to evaluate the duration of rTMS should be further validated.

As previous studies have reported, the effects of rTMS extend beyond the “hot spot” area to influence the functional connectivity among distant brain regions (Watanabe et al., 2014; Valero-Cabré et al., 2017). For instance, Watanabe et al. reported that the functional connectivity between multiple regions assessed by fMRI was significantly reduced by the high frequency rTMS (Watanabe et al., 2014). Our results confirmed this finding, as the application of rTMS significantly decreased the functional connectivity between multiple ROI-pairs in most subjects, demonstrating the ability of rTMS to regulate global brain networks. In addition, the significant disruptions observed within the prefrontal areas (LPF, MPF and RPF) suggested the distant prefrontal cortex may be more sensitive to stimulation compared to motor areas, which is partly in line with the finding of a previous study that observed no alteration among motor network during the vertex stimulation (Jung et al., 2016). Furthermore, the suppressed connections between RPF, LPF and SMA regions imply that rTMS seemed to modify connectivity between symmetric cortical regions in our stimulation protocol. A previous fNIRS-based functional connectivity study reported a symmetrical correlation between the two hemispheres in healthy controls, which represents a regular brain connectivity pattern during resting state (Mesquita et al., 2010). In our study, the alterations in connectivity between symmetrical ROI pairs induced by rTMS may be used to evaluate the efficiency of the rTMS treatment in a clinical setting. For example, potential clinical applications include using rTMS and fNIRS to assess the alterations in patterns of brain activity and interhemispheric connectivity that accompany specific brain disorders (e.g., mental disorder or stroke). In this context, the degree of the characterized activity and connectivity changes could be used to determine the optimal settings of the TMS treatment for specific patients. Similarly, fNIRS and rTMS may be applied to better understand the underlying mechanism of disease progression and monitor the progress of recovering patients.

While the present study provides important details regarding the effects of TMS application on regional blood flow and connectivity, one limitation is that a sham group was not recruited in the experiment, making it difficult to validate if the alterations of hemodynamic response and the connectivity were specifically associated with the rTMS. However, because the hemodynamic response from the sham group has been repeatedly confirmed in previous studies (Thomson et al., 2011a,b; Park et al., 2017), we believe the observed abnormal hemodynamic response could very likely be caused by the rTMS. Considering that the limited sample size in this article may have been insufficient to draw a solid conclusion for its clinical value, we will include a sham group and more subjects in the future to validate the current findings and the feasibility of translating the proposed approach to clinical applications. Additionally, due to a limited number of optodes, in this study our fNIRS device was not able to provide full head coverage to measure comprehensive hemodynamic response signals. It is therefore suggested that future research should utilize an improved setup with more fNIRS optodes. It should also be noted that fNIRS is only able to measure the hemodynamic response on the cortical surface (1–3 cm) due to the inherent limited penetration depth; it would thereby be difficult to investigate any potential hemodynamic changes in subcortical or deep brain areas.

## Conclusion

Global alterations in the hemodynamic response and connectivity between multiple brain regions induced by sub-threshold 10 Hz rTMS were investigated in this study. The HbO concentration in most regions was found to decrease after the onset of the stimulation and gradually recover to the baseline as stimulation continued. Results also showed that rTMS modified the functional connectivity within the prefrontal area and between symmetrical ROI-pairs. Results demonstrated the feasibility of using the combination of rTMS and fNIRS to evaluate both cortical excitability and connectivity, which may pave the way for optimized, patient-specific rTMS treatment programs.

## Author Contributions

RL, JW, ZS and YZ: study design. RL, CW and ZS: data acquisition. RL, TP, and LY: analysis and interpretation. RL, TP, RC, and YZ: manuscript drafting. RL, TP, JW, ZS, CW, LY, RC, and YZ: final approval.

## Conflict of Interest Statement

The authors declare that the research was conducted in the absence of any commercial or financial relationships that could be construed as a potential conflict of interest.
